# P4 ATPases: Flippases in Health and Disease

**DOI:** 10.3390/ijms14047897

**Published:** 2013-04-11

**Authors:** Vincent A. van der Mark, Ronald P.J. Oude Elferink, Coen C. Paulusma

**Affiliations:** Tytgat Institute for Liver and Intestinal Research, Academic Medical Center, Meibergdreef 69-71, 1105 BK Amsterdam, The Netherlands; E-Mails: r.p.oude-elferink@amc.uva.nl (R.P.J.O.E.); c.c.paulusma@amc.uva.nl (C.C.P.)

**Keywords:** P-type ATPase, P4 ATPase, flippase, phospholipid, membrane asymmetry, vesicular transport, disease, ATP8B1, CDC50

## Abstract

P4 ATPases catalyze the translocation of phospholipids from the exoplasmic to the cytosolic leaflet of biological membranes, a process termed “lipid flipping”. Accumulating evidence obtained in lower eukaryotes points to an important role for P4 ATPases in vesicular protein trafficking. The human genome encodes fourteen P4 ATPases (fifteen in mouse) of which the cellular and physiological functions are slowly emerging. Thus far, deficiencies of at least two P4 ATPases, ATP8B1 and ATP8A2, are the cause of severe human disease. However, various mouse models and *in vitro* studies are contributing to our understanding of the cellular and physiological functions of P4-ATPases. This review summarizes current knowledge on the basic function of these phospholipid translocating proteins, their proposed action in intracellular vesicle transport and their physiological role.

## 1. Phospholipid Asymmetry in Biological Membranes

In 1925, the scientists Gorter and Grendel were the first to demonstrate that the plasma membrane of the erythrocyte was a bilayer of phospholipids [1]. From their experiments in “chromocytes of the blood” (*i.e.*, erythrocytes) they concluded that the surface area of lipids extracted from erythrocytes was about twice the surface area of the cells themselves. Gorter and Grendel concluded that “chromocytes are covered by a layer of fatty substances that is two molecules thick” [1]. Indeed, biological membranes are an intricate mixture of many different lipid species that are organized as two back-to-back facing leaflets of phospholipids with hydrophilic head groups facing the hydrophilic environment and hydrophobic acyl tails facing the core of the bilayer [2]. Eukaryotic cell membranes are composed of glycerophospholipids (~65 mol%), cholesterol (~25 mol%) and sphingolipids (~10 mol%) [2,3]. The main structural phospholipids are phosphatidylcholine (PC), phosphatidylserine (PS), phosphatidylethanolamine (PE) and phosphatidylinositol (PI). Most glycerophospholipids are synthesized in the endoplasmic reticulum (ER), while sphingolipids are synthesized in the Golgi. Cardiolipin, a glycerophospholipid exclusively localized to the inner membrane of mitochondria, is manufactured in the mitochondrion. Each leaflet has a different composition which also varies from organelle to organelle. For example, the plasma membrane contains the highest percentage of PS (~10 mol%) as opposed to late endosomes (~2 mol%) or mitochondria (~1 mol%) [3].

One of the hallmarks of eukaryotic membranes in the secretory and endocytic pathways is the asymmetric distribution of the different phospholipid species over the exoplasmic- and cytoplasmic leaflet of the bilayer. Maintaining and dissipating the non-random distribution of phospholipids is crucial for normal regulated membrane (protein) function. For instance, PS exposure at the plasma membrane is an important signal both in the recognition and phagocytosis of apoptotic cells and in the activation of blood coagulation [4]. In the cytosolic leaflet of the plasma membrane, PS is important for the recruitment of protein kinase C (PKC), Src, Ras and Rho proteins via binding to their C2 domain or other cationic domains [5,6].

In the early 1970s, Bretscher, Gordesky and Marinetti demonstrated that the aminophospholipids PS and PE are concentrated in the cytoplasmic leaflet of the erythrocyte bilayer [7,8]. In addition, Verkleij *et al.* showed that PC was equally distributed over both leaflets, whereas sphingomyelin (SM) almost exclusively localized to the exoplasmic leaflet [9]. Less abundant phospholipids, like PI (and derivatives of PI) and phosphatidic acid (PA) are mostly confined to the cytoplasmic leaflet [10–12]. Cholesterol can partition equally and rapidly (milliseconds to seconds) between the two leaflets [13]. It may be enriched in the exoplasmic leaflet due to its high affinity for SM [14], although fluorescent sterols have been predominantly localized to the cytosolic leaflet in Chinese Hamster Ovary cells [15]. Differences in membrane lipid composition likely alter the chemical stability of cholesterol leading to differences in cholesterol distribution, as discussed in [16]. Phospholipids tend to equilibrate between the two leaflets, a process called “scrambling”, which is very slow and depends on lipid head group structure and polarity, as well as hydrophobicity and saturation of the acyl chains and lipid packing in the bilayer [17]. For instance, when the phosphatidyl head group is removed from the glycerol backbone of phosphatidylthioglycerol (a phosphatidylglycerol analog) and is subsequently replaced by dioleoylthioglycerol (a diacylglycerol analog), its unaided translocation rate was decreased from eight days to fifteen seconds [18]. However, due to extensive membrane fusion and budding events by intracellular transport vesicles with and/or from the plasma membrane and cell organelles, phospholipid scrambling is accelerated. Consequently, the non-random transbilayer distribution of phospholipids must be actively maintained, which is accomplished by phospholipid translocating proteins termed flippases and floppases. Lipid flippases and floppases are ATP-dependent proteins that confer transbilayer distribution of phospholipids by translocating phospholipids from the exoplasmic to the cytoplasmic leaflet of the bilayer and *vice versa*, respectively [19]. Actual ATP-dependent flipping of PS and PE, but not PC, was first observed in human erythrocytes by determining the distribution of spin-labeled analogs after their incorporation in the outer leaflet [20]. It is presently well-accepted that proteins from the type 4 subfamily (P4) of the P-type ATPase superfamily are essential in the flipping of phospholipids, which is the subject of the current review. The flopping of lipids is accomplished by members of the ATP-binding cassette (ABC) transporter superfamily, which mediate the ATP-dependent transport of a wide variety of compounds across biological membranes, including (short-chain) phospholipids, sterols and (very long-chain) fatty acids. Apart from ATP-dependent floppases and flippases, the membrane contains ATP-independent bidirectional scramblases that support the Ca^2+^-dependent translocation of phospholipids between leaflets [4,21,22]. Scrambling is of physiological importance for processes such as apoptosis, fission and fusion of transport vesicles, sperm capacitation and intracellular signaling. Lipid flopping and scrambling are not the subject of this review and have been excellently highlighted elsewhere [4,23–25].

## 2. The P4 ATPase Family of Lipid Flippases

**Figure 1:**
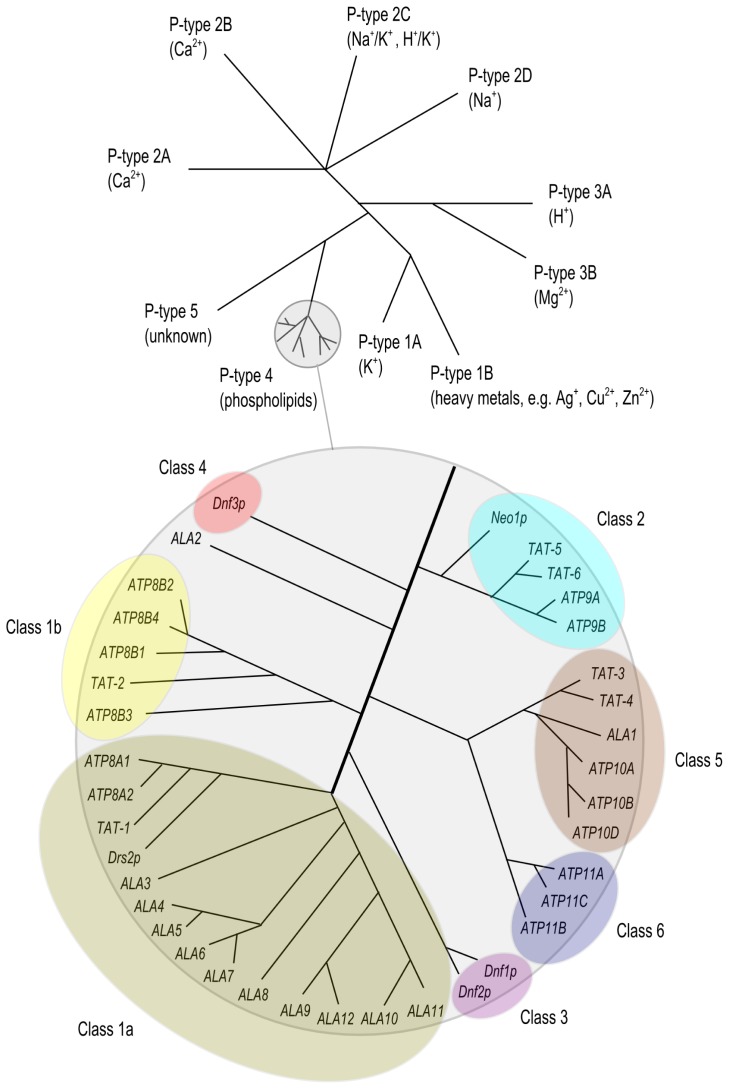
Phylogenetic tree of the P-type ATPase superfamily and of the P4 branch. Substrates of P1–P5 branches are in between brackets. Phylogenetic analyses of the P4 ATPase protein family of mammalian, *A. thaliana*, *S. cerevisiae*, and *C. elegans* is shown and was compiled using ClustalW sequence alignment software [43]. Database accession numbers: *C. elegans*: TAT-1 (NP_001022894), TAT-2 (NP_001023252), TAT-3 (NP_499363), TAT-4 (NP_495244), TAT-5 (NP_001021457), TAT-6 (NP_503858); *A. thaliana*: ALA1 (P98204), ALA2 (P98205), ALA3 (Q9XIE6), ALA4 (Q9LNQ4), ALA5 (Q9SGG3), ALA6 (Q9SLK6), ALA7 (Q9LVK9), ALA8 (Q9LK90), ALA9 (Q9SX33), ALA10 (Q9LI83), ALA11 (Q9SAF5), ALA12 (P57792); *S. cerevisiae*: Drs2p (P39524), Dnf1p (P32660), Dnf2p (Q12675), Dnf3p (Q12674), Neo1p (P40527). *H. sapiens*: ATP8A1 (P70704), ATP8A2 (P98200), ATP8B1 (O43520), ATP8B2 (P98198), ATP8B3 (O60423), ATP8B4 (Q8TF62), ATP9A (O75110), ATP9B (O43861), ATP10A (O60312), ATP10B (O94823), ATP10D (Q9P241), ATP11A (P98196), ATP11B (Q9Y2G3), ATP11C (Q8NB49).

**Figure 2:**
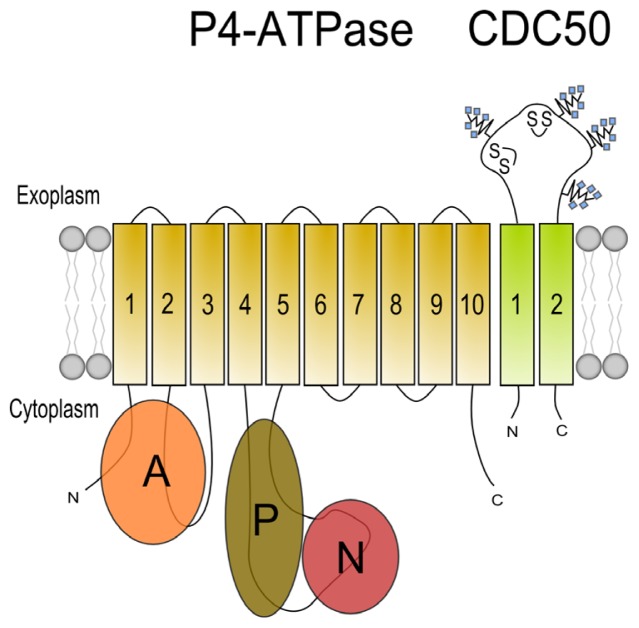
Simplified topological model of a P4 ATPase and its CDC50 subunit. P4 ATPases consist of an actuator (**A**), phosphorylation (**P**), nucleotide binding (**N**) and 10 predicted membrane spanning helices. CDC50 subunits consist of 2 membrane spanning domains with a large extracellular loop containing four possible N-linked glycosylation sites and two disulfide bridges. Modified from Coleman *et al.*[44].

The P-type ATPase superfamily is a large and evolutionary conserved family of proteins of which members are widely expressed in both prokaryotes and eukaryotes. P-type ATPases are integral membrane proteins which, in most cases, mediate the ATP-dependent transport of small cations across biological membranes, including those of intracellular organelles. Based on phylogenetic analysis, this large family is divided into 5 subfamilies (P1-P5), each unique in their class of transport substrates and in subfamily-specific sequence motifs [26] (Figure 1). For instance, the P2 subfamily includes well known proteins including the Ca^2+^ ATPase [27] and the Na^+^/K^+^ ATPase [28], which have been extensively characterized for their molecular activity and (patho) physiological functions; in addition, crystal structures have been obtained for these P-type ATPases [29]. The P4 subfamily members are exclusively expressed in eukaryotic cells and are deviant from the other P-type ATPase subfamilies in that they mediate the transport of phospholipids instead of cations. The first evidence that P4 ATPases are involved in translocating phospholipids was presented by studies on the *Saccharomyces cerevisiae* P4 ATPase Drs2p. This protein was demonstrated to be involved in the transport of fluorescently (NBD)-labeled analogs of PS and PE across the plasma and Golgi membranes of yeast [30,31]. Since then, P4 ATPases from other species such as *Caenorhabditis elegans*[32], *Mus musculus*[33,34], *Homo sapiens*[35,36], *Leishmania donovani*[37] and *Arabidopsis thaliana*[38,39] have been shown to flip NBD- or spin-labeled phospholipids or to be involved in the transbilayer distribution of endogenous phospholipids [36,40–42]. Like all other P-type ATPases, P4 ATPases are multiple transmembrane-spanning proteins with a large cytoplasmic loop harboring the aspartic acid residue which is essential in the reaction cycle of the protein, and both *N*- and *C*-terminal tails protruding into the cytoplasm (Figure 2). The mechanism via which substrates are pumped is based on the autophosphorylation of the invariant aspartic acid residue, which drives the transport cycle of these proteins, hence the name P-type ATPase. At present, none of the P4 ATPases have been crystallized.

## 3. Beta Subunits for P4 ATPases

Similar to other P-type ATPases such as the Na^+^/K^+^- and gastric H^+^/K^+^-ATPases [45,46], P4 ATPases require a heterodimeric interaction with a β-subunit in order to function properly. These β-subunits are glycosylated 50–60 kDa transmembrane proteins of the evolutionary conserved family of CDC50 proteins present in yeast, plants, mammals and *Leishmania*[47]. CDC50 proteins have two putative transmembrane domains and a large loop that protrudes into the exoplasmic space (Figure 2). Although they do not show sequence similarity to the Na^+^/K^+^-ATPase β- and γ-subunits, they do display structural and functional similarities [48]. For the Na^+^/K^+^-ATPase is has been shown that assembly of the α- and β-subunits in the ER is essential for α-subunit maturation, ER exit, subcellular trafficking and modulation of α-subunit activity [49,50]. Protein sorting signals are present in both the α- and β-subunits [51,52]. Similarly, CDC50 protein-P4 ATPase heterodimerization is pivotal for release of the P4 ATPase from the ER [36,53–56]. Although the contribution of CDC50 β-subunits to subcellular trafficking of the P4 ATPase is not completely clear, Lopez-Marques *et al.* have demonstrated for plant P4 ATPases that it is the P4 ATPase rather than the β-subunit that determines the subcellular localization [38]. Similarly, these authors showed that the substrate specificity is determined by the P4 ATPase rather than by the β-subunit. Indeed, in a very elegant study, Baldridge and Graham have identified amino acid residues in the yeast P4 ATPases Drs2 and Dnf1, involved in the flipping of PS and PC, respectively, which determine these substrate specificities [57]. Using an exhaustive number of chimeric Drs2 and Dnf1 proteins that were analyzed for their ability to translocate PC or PS, they identified specific amino acid residues in transmembrane domains (TM) 1–4 of both P4 ATPases that determined substrate preferences. They ultimately showed that substituting a tyrosine for a phenylalanine at residue 618 in TM4 of Dnf1 resulted in the flipping of PS, whereas substitution of the reciprocal phenylalanine for a tyrosine at amino acid 511 in TM4 abrogated PS recognition of Drs2. These data indicate that it is not the β-subunit that determines the substrate specificity, but the P4 ATPase itself.

In mammals, three CDC50 proteins are expressed that are termed CDC50A-C [58,59]; CDC50A and CDC50B are ubiquitously expressed while CDC50C is predominantly expressed in testis and brain [58]. Since the human genome encodes fourteen P4 ATPases (fifteen in mice [60]) and only three CDC50 proteins, this indicates that one CDC50 protein can interact with multiple P4-ATPases. Indeed, co-immunoprecipitation studies have shown that CDC50A, which is the most abundantly-expressed CDC50 protein, can interact with eleven out of fourteen P4 ATPases, whereas CDC50B interacted with at least two P4 ATPases [36,56,61]. ATP8B3, which is highly expressed in the testis, does not interact with CDC50A or CDC50B, and may heterodimerize with CDC50C [56]. Interestingly there are several P4 ATPases that do not interact with any of the CDC50 proteins; ATP9A, ATP9B and the yeast orthologue Neo1p [61,62]. It is possible that these P4-ATPases do not require a subunit for correct trafficking or activity. It remains to be determined which endogenous heterodimers are expressed in which tissues and cell types. Coleman and Molday showed that endogenous CDC50A and ATP8A2 are present as a heterodimer in photoreceptor disc membranes [41].

## 4. The Reaction Cycle

**Figure 3:**
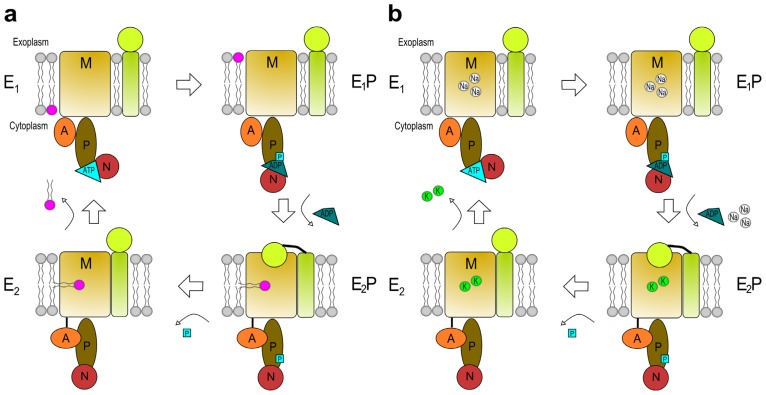
Proposed reaction cycles of a P4 ATPase (**a**) and a P2C ATPase (Na^+^/K^+^ ATPase) (**b**) complexed with their subunit. P-type ATPases cycle through four main separate conformations when transporting ligands. In the E1 state the P-type ATPases have high affinity for intracellular ligands; Na^+^ in the case of the Na^+^/K^+^ ATPase, unknown or none for the P4 ATPase. Binding of ATP to the N-domain and subsequent phosphorylation of the P domain results in the E1-P state. While converting from E1-P to E2-P, intracellular ligands (3 Na^+^ for the Na^+^/K^+^ ATPase) are released into the exoplasmic milieu and the A-domain rotates. This allows binding of extracellular ligands (2 K^+^ for the Na^+^/K^+^ ATPase) or a phospholipid (depicted in pink) from the exoplasmic leaflet. Affinity for the subunit is highest in this state and this interaction may assist in binding of the phospholipid. Dephosphorylation changes the enzyme from the E2-P to the E2 state. Movement of the A-domain away from the P-domain reverts the ATPase back to the E1 state thereby translocating the extracellular ligands or the phospholipid to the cytoplasmic side. Adapted from Coleman *et al.* and Lenoir *et al.*[44,65].

It is presently not clear how these P4 ATPases transport phospholipids across lipid bilayers [63]. However, apart from a role in stabilization of the P4 ATPase and ER exit, there is experimental evidence to support a role for CDC50 proteins in the reaction cycle of the P4 ATPases Drs2p, ATP8B1, ATP8B2 and ATP8A2 [41,53,64,65]. Coleman and Molday demonstrated PS flipping activity upon reconstitution of CDC50A and ATP8A2 into liposomes [41]. For the purified Cdc50p/Drs2p heterodimer it was found that the β-subunit prefers to bind the E2-P conformation of the P4-ATPase (explained in the next paragraph and Figure 3a) [53,65]. Based on these findings, Stone and Williamson proposed that the phospholipid could be loaded on the P4 ATPase/CDC50 heterodimer in the E2-P conformation, which is exposed on the luminal side of the bilayer [66].

It has been suggested that the reaction cycle is analogous to those of other established P-type ATPases [26], *i.e.*, the classic Post-Albers or E1E2 model [67,68] (Figure 3a,b). In the E1 unbound conformational state cytosolic ligands can easily bind to the P-type ATPase. Ligand binding (such as Na^+^ for the Na^+^/K^+^-ATPase) facilitates interaction of Mg^2+^-ATP with the nucleotide binding domain (N); however, for P4 ATPases it is elusive whether there are intracellular ligands required to initiate the reaction cycle. Binding of the substrate to be transported and of ATP, results in phosphorylation of the conserved aspartate in the phosphorylation domain (P) [64]. This autophosphorylation introduces a small conformational change in the protein generating the E1-P state. Release of ADP, movement of the P-domain stretching the link between TM-3 and the actuator domain (A) and rotation of the A-domain generates a large conformational change transforming the protein into the E2-P state [29]. During this conversion affinity for cytosolic bound ligands is decreased granting their release at the exoplasmic side. Exoplasmic ligands are now able to bind to a high affinity region in the membrane domain (M): K^+^ binds to the Na^+^/K^+^-ATPase and a phospholipid from the outer membrane leaflet to the P4 ATPase. In the E2-P state the Na^+^/K^+^-ATPase is stabilized by its β-subunit [69], while the P4 type ATPase has its highest affinity for its CDC50 subunit, possibly via interactions of the transmembrane regions and/or the subunit’s large exoplasmic loop [65]. The CDC50 subunit assists in binding of the phospholipid to the P4 ATPase, opens up a pathway for translocation or occludes the bound phospholipid, but, together with the bound extracellular ligand, is likely an important component in dephosphorylation of the E2-P state to the E2 state [65]. The transition from E2 to E1 occurs when the A-domain returns to its original position away from the P-domain. Affinities for the subunit and exoplasmic ligands are decreased resulting in the release of ligands towards the cytoplasmic side.

Other protein and lipid factors are probably involved in completing the reaction cycle of the complex. In yeast, phosphatidylinositol-4 phosphate (PI4P) synthesis by the phosphatidylinositol-4 kinase (PI4K) Pik1 and binding of PI4P to the *C*-terminal tail of Drs2p were required for flippase activity [64,70]. Dephosphorylation of the Drs2p-Cdc50p complex in crude membranes in the presence of PS only occurred when PI4P was present as well [64]. Direct regulatory phosphorylation of yeast P4-ATPases occurs via the serine/threonine protein kinases (Fpk) 1 and 2 [71]. Cells lacking these kinases are deficient in (NBD-labeled) phospholipid uptake.

The E1E2 model provides an explanation for the pumping mechanism of P4 ATPases, but does not take the “giant substrate problem” into account: compared to an ion, a phospholipid with its large apolair acyl chains is a large, bulky substrate that might prove too large to fit in its proposed binding pocket in its entirety. The “credit card model” provides a way to circumvent this problem; only the charged phospholipid head group is bound to the P4 ATPase while the hydrophobic tails project out of the protein and remain within the lipid bilayer during the translocation process [57]. It is of interest to mention that the amino acids responsible for H^+^ binding and transport in H^+^-ATPases of the P3A subfamily (*i.e.*, a conserved aspartic acid and arginine residue) form a water-filled space inside the transporter big enough to contain a phospholipid head group [66,72]. In analogy to this transport mechanism, Coleman *et al.* presented evidence to suggest a similar mode of transport for PS by the P4 ATPase ATP8A2 [42].

In an extensive screen for residues in TM1-6 in Drs2p and Dnf1p Baldridge and Graham identified two clusters of residues involved in phospholipid selection [73]. One of these clusters is located in TM1 and 2 on the exoplasmic side of the membrane and is termed the “entry gate”; important for PS recognition in Drs2p and PC recognition in Dnf1p. The other cluster, termed the “exit gate”, is located in TM3 and 4 near the cytosolic side of the membrane and is proposed to be involved in selection of the phospholipid before or during the E2-P to E2 transition. This two-gate mechanism thus proposes alternative binding sites for the phospholipid at the membrane facing surfaces of P4-ATPases.

## 5. P4 ATPases and Vesicular Transport

Accumulating evidence obtained in yeast, worms and plants point to an important role for P4 ATPases in the biogenesis of intracellular transport vesicles (reviewed in [47,74]). Vesicular trafficking is a continuous cellular activity in which cells recycle a membrane area equivalent to their cell surface one to five times per hour by endocytic activity alone [75]. P4 ATPases are implicated in the initiation of vesicle biogenesis. P4 ATPase-mediated flipping of a phospholipid could result in a local increase in the concentration of phospholipids in the cytoplasmic leaflet of the lipid bilayer. In turn, this will generate a cytoplasmic facing membrane curvature, which can be explained by the “bilayer couple hypothesis” [76]: a sufficiently large local expansion of the number of phospholipids in one leaflet of a lipid bilayer relative to the opposite leaflet will create an increased area and force the coupled leaflet to minimize its energy state and maintain its hydrophobic interactions between the leaflets and thereby induces local bending of the bilayer. Farge *et al.*[77] have shown that exogenous addition to human erythroleukemia K562 cells of the aminophospholipids PS and PE, which are flipped to the cytosolic leaflet (resulting in an increase of the cytosolic surface area), causes an increase in cellular endocytosis. In contrast, addition of the non-flippable PS analog lyso-alpha-phosphatidylserine does not cause an increase in endocytosis; an observation that supports the “bilayer couple hypothesis”. Thus, the induction of membrane curvature, possibly mediated by P4 ATPases, is the initiating event in the generation of transport vesicles e.g. at the *trans*-Golgi network (TGN) or at the plasma membrane. Also, a local increase in PS and/or PE concentration in the cytosolic leaflet of the membrane can provide a docking platform for curvature-stabilizing and vesicle-forming proteins [78]. Alternatively, membrane curvature can be induced by insertion of hydrophobic proteins, through tension generating protein scaffolds such as BAR-domain proteins or force transmission from SNARE complexes [79]; whether P4 ATPases or BAR domain proteins act alone or whether it is a collaborative effort of these type of proteins to induce initial curvature is presently not known. Either way, membrane curvature is essential for the biogenesis of transport vesicles as it creates a binding scaffold for various coat proteins e.g. clathrin, COPI or COPII, which induces the mobilization of the vesicle-generating protein machinery [80–84].

*S. cerevisiae* expresses five P4 ATPases which all are involved in the biogenesis of intracellular transport vesicles in the biosynthetic and endocytic pathways [85]. For the most extensively studied Drs2p it has been demonstrated that it is involved in the initiation of clathrin-coated vesicle biogenesis [85–87]. Drs2p, which physically interacts with adaptor protein-1 (AP-1) [87], shuttles between the TGN and early endosomes in an AP-1/clathrin-dependent pathway. Mutant *drs2*Δ cells are impaired in clathrin-coated vesicle formation; however, Drs2p is not essential for clathrin recruitment since in *drs2*Δ mutant cells clathrin and AP-1 are normally localized in the TGN [62,86,88]. Importantly, Drs2p activity is essential for the formation of clathrin-coated vesicles, possibly by concentrating phospholipids in the cytosolic leaflet of the TGN. Thus, Drs2p-mediated lipid flipping may induce membrane curvature and drive the formation of a clathrin-coated vesicle at the TGN or endosome. In addition to AP-1, Drs2p interacts with many more proteins of the vesicle trafficking machinery. For instance, Drs2p interacts with the F-box protein Rcy1, which is involved in recycling between the endosomes and TGN [54], but Drs2p also forms a ternary complex with the Arf guanine nucleotide-exchange factor Gea2p and the Arf-like protein Arl1 in order to regulate its flippase activity in the Golgi [70,89,90]. Genetic associations with *drs2* have been described for ADP-ribosylation factors (recruitment of coat protein complexes) and Rab proteins [54,90–92]. Using tandem-affinity purification and mass spectrometric analyses, Puts *et al.* have identified additional Drs2p-interacting proteins with a role in vesicular trafficking [91]. Amongst others, three proteins involved in phosphoinositide metabolism were identified, including a PI4P phosphatase. Phosphoinositides are critical regulators of membrane and protein trafficking, mainly via binding and activation of downstream target proteins [93]. PI4P was shown to be essential for the recruitment of the clathrin coat protein machinery to the TGN, and thus for the initiation of clathrin-coated vesicle generation [94,95]. Natarajan *et al.* have shown that interaction between Drs2p and PI4P is essential to catalyze NBD-PS flipping across isolated TGN membranes [70]. All these observations suggest that Drs2p-catalyzed phospholipid flipping is the initiating event in the biogenesis of clathrin-coated vesicles and the mobilization of the vesicle-trafficking protein machinery in the TGN. In addition, the different subcellular distributions of the newly identified Drs2p-interacting proteins (*i.e.*, Golgi apparatus (GA), ER, plasma membrane, and vacuole) suggest that Drs2p resides in different protein complexes within distinct trafficking pathways [91].

In yeast, the plasma membrane-associated P4 ATPases Dnf1p and Dnf2p are important in the biogenesis of endocytic transport vesicles. *Dnf1*Δ*dnf2*Δ double mutant cells have a defect in the internalization step of fluid-phase endocytosis, *drs2*Δ*dnf1*Δ double mutant cells are impaired in the TGN-directed transport of alkaline phosphatase while *dnf1*Δ*dnf2*Δ*drs2*Δ triple mutants are deficient in receptor-mediated endocytosis [62,96,97]. Importantly, the phenotypes of the *dnf1*Δ*dnf2*Δ and *dnf1*Δ*dnf2*Δ*drs2*Δ mutants coincided with plasma membrane exposition of small amounts of natural PE and PS [97]. These observations justify the speculation that Dnf1p and Dnf2p flip glycerophospholipids to initiate membrane vesiculation. Alder-Baerens, *et al.* have shown that Dnf3p in isolated post-Golgi secretory vesicles is involved in the flipping of NBD-PS, PE, and PC [98]. Finally, Neo1p, the only essential P4 ATPase in yeast, is implicated in the retrograde, COPI-dependent transport between Golgi apparatus and ER [99,100].

Besides a role in intracellular trafficking, P4-ATPases may also be important for plasma membrane remodeling. Dnf1p- or Dnf2p-Lem3p heterodimer-mediated flipping of PE from the exoplasmic to the cytosolic leaflet is required for the dissociation of Cdc42 from the polar cortex [101]. The Cdc42 GTPase is a key regulator of cell polarity in yeast via its role in the regulation of actin polymerization. The release of Cdc42 from the cortex, which leads to a blockage of actin polymerization, triggers the growth switch of the growing bud tip of the daughter cell. Cdc42 release is initiated by locally increasing the concentration of uncharged PE *versus* charged PS, which results in the disruption of the charge interaction of Cdc42 with the cytosolic leaflet of the plasma membrane. Alternatively, one may speculate that Cdc42p is released from the membrane via endocytosis, initiated by a P4 ATPase-mediated increase in local concentration of PE in the cytosolic leaflet of the bilayer.

In the plant *Arabidopsis thaliana*, the P4 ATPase ALA3 is involved in phospholipid flipping and vesicle biogenesis [39]. *Ala3* mutant plants display impaired growth and have a defect in the secretion of TGN-derived vesicles, which contain enzymes required for cell wall breakdown and release of the peripheral cell layer. This process is required for growth of roots and shoots.

Also in *C. elegans* P4 ATPases are involved in vesicle transport. The P4 ATPase TAT-1 has been implicated in the early steps of endocytosis in intestinal epithelial cells and oocytes and in the biogenesis of lysosomes [102]. In another study both the *C. elegans* Cdc50 β-subunit CHAT-1 and TAT-1 itself are shown to be important in maintaining normal endocytic recycling by promoting membrane tubulation of the early endosome [32]. Recently, a role for the P4 ATPase TAT-5 was suggested in the regulation of ectosome shedding, a process that is relevant for the cellular excretion of proteins, RNA and miRNA and critical for the modulation of cellular processes [103].

In Chinese hamster ovary cells the ATP8A1-CDC50A heterodimer is important for cell migration [104]. Cell motility occurs through reorganization of cortical actin filaments at the leading edge, which subsequently move the plasma membrane forward. In contrast to the P4 ATPase-mediated PE internalization and subsequent release of Cdc42 from the growing bud tip of the yeast cell [101], ATP8A1 activity may provide a docking platform for actin polymerization proteins such as Rac1, which localizes to membrane ruffles, and which localization is diminished in ATP8A1 depleted cells. The authors showed that absence of Rac1 mobilization coincides with impaired plasma membrane PE internalization and suggests that ATP8A1 is involved in the generation of membrane ruffles resulting in cell motility.

**Figure 4:**
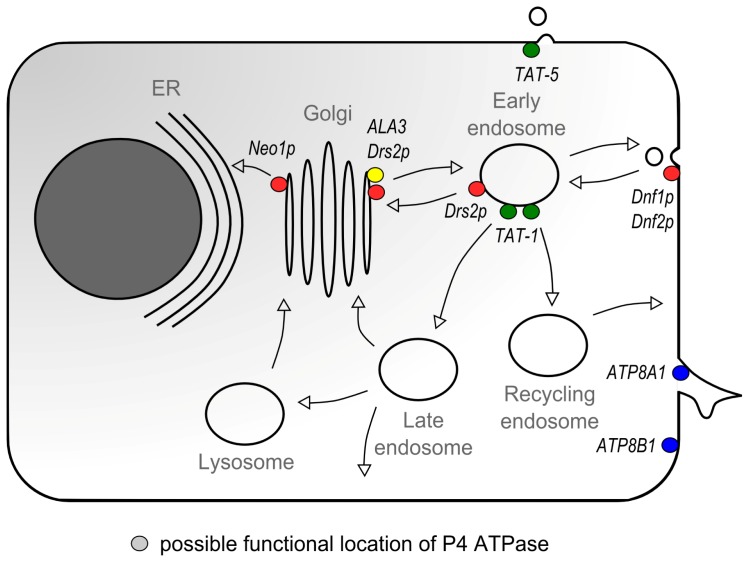
Proposed roles of P4 ATPases in intracellular vesicle trafficking routes in *S. cerevisiae*, *A. thaliana* and *C. elegans*. Although many P4 ATPases are linked to intracellular trafficking defects, only a few have been specifically linked to certain organelles. *S. cerevisiae* Neo1p has been implicated in retrograde, COPI-dependent trafficking from the Golgi to the ER [99,100]. Drs2p in yeast is involved in the formation of AP-1/clathrin coated vesicles back and forth between the TGN and early endosomes [40,54,62,86,87]. Yeast Dnf1p and Dnf2p play a role in the formation of endocytic vesicles [62,96,97]. *A. thaliana* ALA3 is necessary for the synthesis of secretory vesicles from the TGN [39]. *C. elegans* TAT-1 is important in maintaining normal endocytic recycling and biogenesis of lysosomes [32,102] whereas TAT-5 was suggested to be involved in the regulation of ectosome shedding [103]. In Chinese hamster ovary cells ATP8A1 plays a role in cell migration by assisting in the formation of plasma membrane ruffles [104]. ATP8B1 is necessary for apical hepatocyte membrane integrity in *M. musculus*[108,109,126]. Possible functional locations of P4-ATPases are represented by colored circles; red for *S. cerevisiae*, yellow for *A. thaliana*, green for *C. elegans* and blue for mammalian P4-ATPases. See text for further details.

All these studies indicate that P4 ATPases are an important driving force in the initiation of the formation of transport vesicles in multiple parts of intracellular trafficking pathways (Figure 4). It seems likely that P4 ATPase-mediated flipping causes an initial local membrane curvature that provides a scaffold for binding of proteins of the vesicle generation machinery.

## 6. The (Patho) Physiological Function of Mammalian P4 ATPases

In contrast to lower eukaryotes, much less is known about the cellular and physiological functions of mammalian P4 ATPases. The mammalian P4 ATPase subfamily consists of fourteen proteins (fifteen in mice) of which presently two are implicated in protein trafficking. Thus far, one P4 ATPase has been associated with human disease; however, various *in vitro* studies and studies in mouse models are contributing to our understanding of the cellular and (patho) physiological functions of mammalian P4 ATPases, and will be highlighted below. The pathophysiological characteristics of P4 ATPase deficiencies in mice and humans are summarized in Table 1.

*Atp8a1* knockout mice are characterized by impaired hippocampus-dependent learning (assessed by water-avoidance experiments) and increased activity (assessed by open field testing). These behavioural problems coincided with increased PS externalization in hippocampal neurons [105]. Interestingly, *Atp8a1*^−/−^ erythrocytes displayed no PS exposure on the exoplasmic leaflet, which, according to the authors, was most likely due to compensatory expression of ATP8A2 in these cells. ATP8A1 is present in erythrocyte precursors and present on the membrane of mature erythrocytes and has a PS flippase activity [125]. Taken together with the compensatory expression of ATP8A2 in *Atp8a1*^−/−^ mice, both ATP8A1 and ATP8A2 are prime candidates for the ATP-dependent aminophospholipid translocase activity that was first discovered in erythrocytes [20].

Wabbler-lethal mutant mice display neurodegenerative disease and axonal degeneration in the central and peripheral nervous system. Zhu *et al.* identified *Atp8a2* as the causative gene in these mice which are growth retarded and do not survive beyond 4 months of age [107]. Mutant animals display central chromatolysis in neuronal cells, a characteristic of axon dystrophy without cell loss. Axonal transport of phosphorylated neurofilaments is disturbed in the lumbar motor neurons, indicating a role for ATP8A2 in the vesicular, axonal transport of this protein. These *in vivo* data suggest a role for ATP8A2 as a PS flippase in the maintenance of axon polarity. *In vitro* data obtained in neuronal PC12 cells and rat hippocampal neurons also indicate a role for ATP8A2 (in conjunction with its β-subunit CDC50A) in promoting neurite outgrowth [127]. Indeed, a mutated *ATP8A2* was identified in a patient with severe mental retardation and decreased muscle tone (hypotonia) [128]. However, screening of an additional 37 patients with a similar phenotype did not result in the identification of *ATP8A2* mutations. Additionally, a recessive missense mutation in *ATP8A2* was detected in three members of a consanguineous family affected with cerebellar ataxia, mental retardation and disequilibrium syndrome (CAMRQ) [106]. Although only a small number of patients have been identified with mutations in *ATP8A2* it seems likely that it is a risk factor for neurodegenerative diseases.

In mice, ATP8B3 is implicated in sperm cell acrosome formation and capacitation [113,114]. The acrosome is a Golgi-derived organelle in the head of the sperm cell that contains digestive enzymes. Upon fertilization, the acrosome fuses with the sperm cell membrane to release, amongst others, hydrolases that digest the zona pellucida of the oocyte, after which the sperm cell can fuse with the oocyte. Capacitation is a cascade of membrane remodelling events in sperm cells to prepare them for penetration of the zona pellucida. Capacitation is associated with PS exposure in the sperm cell head. In contrast to control cells, *Atp8b3*^−/−^ cells exposed PS in the outer membrane leaflet even before capacitation. Although the *in vitro* fertilization capacity of *Atp8b3*^−/−^ sperm cells was reduced (due to reduced rate of zona pellucida penetration), litter sizes were not significantly reduced [114]. Besides ATP8B3, mice express ATP8B5 (also termed FetA) in the acrosomal membrane of mature and developing sperm cells [60]. Knockdown of *Atp8b5* in a mouse mastocytoma cell line showed profound effects on Golgi structure and protein secretion. Although presently unclear, ATP8B5 may (partially) compensate for the loss of ATP8B3 expression in the acrosomal membrane.

A significant association between Alzheimer’s disease and the *ATP8B4* locus on chromosome 15 was reported [115]. One of the single-nucleotide polymorphisms (SNPs) described localized close to the *ATP8B4* gene. Although a follow-up on this association never occurred, this observation might suggest that mutations in *ATP8B4* may predispose to Alzheimer’s disease.

Mice heterozygous for the P4-ATPase *Atp10a* (also named *Atp10c*) develop insulin resistance, hyperlipidemia and are hyperinsulinemic and provide a model for type-2 diabetes mellitus and diet-induced obesity [116,117]. ATP10A has been implicated in the regulation of insulin-stimulated glucose uptake by plasma membrane mobilization of GLUT4-containing vesicles [116,117,129]. Moreover, mRNA expression levels of a canine *ATP10A* orthologue in visceral adipose tissue were found to be five times higher in obese dogs in comparison with lean dogs, suggesting an involvement of ATP10A in response to diet-induced obesity [130]. It remains to be demonstrated if ATP10A is directly involved in insulin-stimulated mobilization of GLUT4-containing vesicles to the plasma membrane, or whether ATP10A exerts its effects on glucose metabolism via regulation of insulin receptor-induced signalling. Although *ATP10A* has not been identified as a risk gene for type 2 diabetes in Caucasian Europeans [118], the genomic region encompassing *ATP10A* was identified as a risk locus in a genome-wide association study (GWAS) of insulin resistance in an African American cohort [119]. In addition, the CpG methylation state of the *ATP10A* gene has been found to be a good biomarker for prediction of weight-loss of obese or overweight men in response to a hypocaloric diet [120]. A methylation microarray followed by mass spectrometry profiling of specific CpG methylation sites of high and low responders to a hypocaloric diet showed that a higher methylation state of *ATP10A* predicted a decreased response to the diet. This could indicate that an altered epigenomic regulation of *ATP10A* may determine the degree of insulin-resistance in humans.

ATP10D has also been implicated in obesity and hyperinsulinemia in mice. *Atp10d* is mutated (*i.e.*, a premature stop codon in exon 12) in C57BL/6J mice [121], which have a predisposition to develop obesity, hyperglycemia and hyperinsulinemia when placed on a high-fat diet [131]. ATP10D is expressed in kidney and placenta, possibly in macrophages. At present, the relation between these phenotypes and ATP10D deficiency is not known.

Recently, the *ATP11A* gene was identified as a predictive marker for metastasis in colorectal cancer (CRC) [122]. The authors reported that *ATP11A* mRNA levels were significantly elevated in CRC tissue compared to control tissue. Patients expressing high *ATP11A* levels showed reduced, disease-free survival rates, an observation on which the authors concluded that *ATP11A* was a good predictive marker for metastasis in CRC. How ATP11A activity correlates with metastasis in CRC remains elusive.

The X-linked *Atp11c* mutant mouse has been characterized by an arrest in B cell development, conjugated hyperbilirubinemia, unconjugated hypercholanemia, hepatocellular carcinoma, anemia and dystocia [34,123,124]. The relation between these diverse phenotypes and ATP11C deficiency is presently unclear. However, it is speculated that the B cell lymphopenia is caused by a defect in the transition from the pro- to the pre-B cell stage [34]. This transition requires the clathrin-mediated endocytosis of ligand-bound interleukin-7 receptor (IL-7R). ATP11C deficient pro-B cells display higher surface expression of IL7-R, which may be caused by impaired internalization of the IL-7 bound IL7-R and subsequent impaired signalling. ATP11C may be important in the clathrin-mediated endocytosis of the activated IL7-R. In humans, *ATP11C* has been mapped to Xq27, a genomic region associated with X-linked inherited disorders, including hypoparathyroidism, albinism-deafness, and thoracoabdominal syndrome [132]. If and how ATP11C deficiency is associated with these phenotypes remains to be demonstrated.

## 7. ATP8B1 and Human Disease

ATP8B1 is the most extensively studied human P4-Atpase. Mutations in *ATP8B1* cause progressive familial intrahepatic cholestasis type 1 (PFIC1) and benign recurrent intrahepatic cholestasis type 1 (BRIC1) [133,134], two liver disorders which were first described by Clayton *et al.*[135] and Summerskill and Walshe [136] (reviewed in [110]). PFIC1 and the less severe BRIC1 have an onset during the early stages of life and are considered as two ends of a continuum of liver disease, characterized by impaired bile flow (*i.e.*, cholestasis). The exact prevalence of PFIC1 or BRIC1 is unknown, but for PFIC1 is estimated to be 1 in 100,000–900,000 [137,138]. PFIC1 is characterized by fierce pruritis, fat malabsorption, failure to thrive and progressive liver disease leading to fibrosis and cirrhosis. BRIC1 patients suffer from intermittent bouts of cholestasis and pruritis and sustain less pronounced liver damage [139]. Liver transplantation or bile diverting procedures during childhood are necessary for most PFIC1 and BRIC1 patients who develop a chronic cholestasis [140,141]. PFIC1 and BRIC1 patients can also develop extrahepatic disease, such as diarrhea, hearing loss, pancreatitis, rickets, and pneumonia [112,142–144]. There is evidence for clinical heterogeneity in the group of PFIC1 patients, but apart from differences in severity related to the type of mutation, the background of this heterogeneity is not known [134,145]. After liver transplantation or biliary diversion, PFIC1 patients usually suffer from an exacerbated form of diarrhea which can be ameliorated by cholestyramine, a bile salt binding resin. This suggests that the intestine of PFIC1 patients has increased sensitivity for a restoration of bile salt secretion. Interestingly, PFIC1 patients who received a liver transplant develop liver steatosis the etiology of which is unknown [140,142,146].

ATP8B1 is expressed in many tissues, including the liver, pancreas, small intestine, bladder, stomach, and prostate and localizes to the apical membrane of many epithelial cells, including the canalicular membrane of hepatocytes [36,147,148]. Our laboratory has extensively studied the etiology of ATP8B1-associated cholestasis using knock-in mice for a prototypic PFIC1 mutation, a glycine-to-valine substitution at amino acid 308 that results in near complete absence of the protein (G308V) [109,111,149]. We and others have shown that the apical membrane of ATP8B1-deficient hepatocytes is sensitive to bile salt-induced membrane damage evidenced by extraction of membrane components, including cholesterol and PS [108,109]. From our observations we have hypothesized that ATP8B1 is important to reduce the outer leaflet content of PS, so as to increase the relative sphingomyelin content, which together with cholesterol forms a rigid, liquid-ordered membrane that is resistant towards detergents such as bile salts; ATP8B1-deficiency thus leads to loss of the normal phospholipid asymmetry of the canalicular membrane. As a result the canalicular membrane becomes more sensitive to extraction of cholesterol by hydrophobic bile salts, which impairs the activity of the major bile salt transporter ABCB11 and, as a consequence, causes cholestasis [126] (reviewed in [110]). Enhanced biliary cholesterol output in Atp8b1-deficient mice turned out to be independent of the cholesterol transporter ABCG5/G8, since ATP8B1 deficient mice with inactivated ABCG5/G8 also displayed enhanced cholesterol output [150]. In the same mouse model a progressive degeneration of the cochlear hair cells was present, explaining the hearing-loss in PFIC1 and BRIC1 patients [112]. Recently, a role for ATP8B1 in pulmonary cardiolipin uptake was suggested [33]. Using ATP8B1 deficient mice, the authors showed that pulmonary infection in these mice was associated with elevated cardiolipin levels. They concluded that enhanced pulmonary cardiolipin in ATP8B1-deficiency impairs lung and lung surfactant function, and may be the underlying cause of the pulmonary problems observed in some PFIC1 patients. However, it remains unclear whether ATP8B1 is a cardiolipin transporter and whether susceptibility to pneumonia in ATP8B1 deficient patients results from a defect in cardiolipin transport [151].

An alternative hypothesis for the etiology of ATP8B1-associated cholestasis is based on findings that ATP8B1 deficiency impairs farnesoid X Receptor (FXR)-dependent signaling. FXR is a bile salt sensing receptor that regulates bile salt homeostasis in hepatocytes, by inactivating bile salt synthesis and by upregulating *ABCB11*. Using Chinese hamster ovary cells, Caco-2 cells and human hepatocytes, Chen *et al.* showed that ATP8B1 depletion resulted in diminished FXR signaling caused by impaired PKCζ-mediated phosphorylation of cytosolic FXR and subsequent absence of nuclear translocation [152–154]. Inhibition of PKCζ activity coincided with reduced *ABCB11* promoter activation, whereas PKCζ overexpression resulted in activation of *ABCB11* promoter activity. Based on these findings, the authors hypothesized that ATP8B1 activity is essential for PKCζ activation, which leads to FXR phosphorylation, nuclear translocation, and transcriptional regulation of target genes. In addition, transient overexpression of mutant ATP8B1 in HepG2 cells caused a decrease in FXR luciferase reporter activity when compared to wild type ATP8B1 induced activity [155]. Thus far, however, no experimental evidence is published to show that ATP8B1-mediated PS flipping is essential for PKCζ activation. Furthermore and in contrast to Chen *et al.*, Cai *et al.*, using Caco-2 cells and human and rat hepatocytes, have shown that ATP8B1 depletion actually leaves FXR activity unaffected [108].

Other than a crucial role in hepatocanalicular membrane asymmetry, ATP8B1 may also have a role in membrane trafficking and vesicular transport. Verhulst *et al.* observed that knockdown of ATP8B1 in Caco-2 cells led to a loss in microvilli, an unorganized apical actin cytoskeleton and a posttranscriptional defect in apical protein expression [156]. It seems unlikely that a similar dramatic impairment of apical membrane assembly of intestinal epithelial cells also occurs in patients with PFIC1. There is, however, clearly room for a more subtle defect in this process as there is an intestinal phenotype in these patients.

## 8. Concluding Remarks

P4 ATPases serve important functions in cellular physiology. Accumulating evidence obtained in lower eukaryotes point to an important role for P4 ATPases in initiating membrane vesicle biogenesis, which has important implications for P4 ATPases in polarized membrane and protein transport. Still, flipping by P4 ATPases, as of yet, has not been shown to induce membrane curvature directly. Secondly, P4 ATPases are important in maintaining an optimal physical state of the membrane, which is essential for proper membrane barrier and membrane protein function. Obviously, the physiological and cellular functions of most mammalian P4 ATPases are only slowly emerging. Studies in cell lines suggest important functions for P4 ATPases in transport and signalling processes. In organelles where membrane asymmetry has been established (*i.e.*, the late secretory and endocytic pathways), studies in *S. cerevisiae*, *A. thaliana* and *C. elegans* have shown the importance of P4 ATPases in intracellular trafficking. Specifically, most of the P4 ATPases studied seem to be involved in transport between the TGN and early endosomes, endocytosis or endocytic recycling. Live cell imaging studies and tracking of P4-ATPases inside the cell could perhaps reveal an even clearer picture regarding the intracellular pathways they are involved in. Multiple mouse models are presently available and show that P4 ATPases fulfil multiple important physiological functions; deficiencies result in a wide variety of neurological phenotypes, liver disease, immunological problems and type 2 diabetes and diet-induced obesity. How all these distinct phenotypes relate to a defective flippase activity remains to be elucidated.

The existence of a large family of functionally similar flippases suggests there may be a role for redundancy amongst the P4 ATPases. Nevertheless, the mouse models indicate that, if there is redundancy, it is limited as there is room for severe phenotypes. For example; despite the compensatory upregulation of ATP8A2 in ATP8A1 deficient erythrocytes, ATP8A1 deficient mice still present neurological problems. With the exception of ATP8B4 (Alzheimer’s disease) and ATP10A (insulin resistance in African Americans), thus far none of the human P4 ATPases have been identified as risk genes in GWAS. Although future GWAS analyses may reveal P4 ATPases as risk genes, it may be that in humans P4 ATPases encompass critical activities of which deficiencies are embryonic lethal, or that diseases associated with P4 ATPases are just rare (as is the case for PFIC1/BRIC1 disease). Therefore, *in vitro* studies and studies in mouse models will be crucial to identify the cellular and (patho) physiological functions of P4 ATPases and will reveal yet undiscovered P4 ATPase-related diseases.

## Figures and Tables

**Table 1:** Overview of mammalian P4 ATPase deficiencies and their pathophysiological characteristics in mice and humans.

Class	P4 ATPase	Pathophysiology in mice	Pathophysiology in humans	References
1A	ATP8A1	impaired learning, increased physical activity		[105]
	ATP8A2	neurodegenerative disease, axonal degeneration, growth retardation	mental retardation, hypotonia, CAMRQ	[106,107]

1B	ATP8B1	intrahepatic cholestasis, hearing loss	PFIC1, BRIC1	[108–112]
	ATP8B2			
	ATP8B3	sperm capacitation anomalies		[113,114]
	ATP8B4		Alzheimer’s disease	[115]
	ATP8B5		not present in humans	

2	ATP9A			
	ATP9B			

5	ATP10A	insulin resistance, diet-induced obesity, hyperlipidemia, hyperinsulinemia	type 2 diabetes, insulin resistance in African Americans, diet-induced obesity	[116–120]
	ATP10B			
	ATP10D	diet-induced obesity, hyperinsulinemia, hyperglycemia		[121]

6	ATP11A		metastasis in colorectal cancer	[122]
	ATP11B			
	ATP11C	arrested B cell development, dystocia, anemia, hepatocellular carcinoma, conjugated hyperbilirubinemia, unconjugated hypercholanemia		[34,123,124]
